# Diabetes Duration, Cholesterol Levels, and Risk of Cardiovascular Diseases in Individuals With Type 2 Diabetes

**DOI:** 10.1210/clinem/dgae092

**Published:** 2024-02-15

**Authors:** Mee Kyoung Kim, Kyu Na Lee, Kyungdo Han, Seung-Hwan Lee

**Affiliations:** Division of Endocrinology and Metabolism, Department of Internal Medicine, Yeouido St. Mary's Hospital, College of Medicine, The Catholic University of Korea, Seoul 07345, Republic of Korea; Department of Statistics and Actuarial Science, Soongsil University, Seoul 07040, Korea; Department of Statistics and Actuarial Science, Soongsil University, Seoul 07040, Korea; Division of Endocrinology and Metabolism, Department of Internal Medicine, Seoul St. Mary's Hospital, College of Medicine, The Catholic University of Korea, Seoul 06591, Republic of Korea

**Keywords:** duration, cholesterol, cardiovascular disease, diabetes mellitus, Korea

## Abstract

**Objective:**

To investigate the association of diabetes duration with cardiovascular disease (CVD) risk and to examine the relationship between lipid levels and CVD risk over the duration.

**Methods:**

Using the Korean National Health Insurance Service Cohort database, we identified 2 359 243 subjects with type 2 diabetes aged ≥ 20 years in 2015 to 2016. Baseline lipid levels and diabetes duration were evaluated and followed up until December 2020 (mean follow-up, 3.9 years). Subjects were categorized according to diabetes duration (new-onset, < 5 years, 5-9 years, or ≥ 10 years). We analyzed the new-onset diabetes group with low-density lipoprotein cholesterol (LDL-C) < 70 mg/dL as the reference group. The hazard ratios (HRs) and 95% CIs of myocardial infarction (MI) and ischemic stroke (IS) were estimated using a Cox proportional hazards model adjusted for potential confounders.

**Results:**

During follow-up, 45 883 cases of MI and 53 538 cases of IS were identified. The risk of MI or IS began to increase at LDL-C ≥ 160 mg/dL in the new-onset diabetes group, and at LDL-C ≥ 130 mg/dL in the group with diabetes duration < 5 years. Among subjects with diabetes duration of 5 to 9 years, LDL-C levels of 100-129 mg/dL, 130-159 mg/dL, and ≥ 160 mg/dL were significantly associated with the risk of MI (HR [95% CI] 1.13 [1.04-1.22], 1.28 [1.17-1.39], and 1.58 [1.42-1.76], respectively). MI risk in the diabetes duration ≥ 10 years group was increased by 16%, even in the LDL-C 70-99 mg/dL population (HR [95% CI] 1.16 [1.08-1.25]).

**Conclusion:**

This population-based longitudinal study revealed that the LDL-C cutoff level for increasing the risk of CVD varied with diabetes duration and that the target LDL-C level should depend on the duration.

Diabetes mellitus (DM) is only included in the cardiovascular disease (CVD) risk prediction model as a dichotomous factor, such as having diabetes or not, or is only considered based on the presence or absence of target organ damage in DM (1-3). However, not every person with DM experiences the same risk of CVD (4, 5). In recent decades, the age of DM onset has decreased and there is increasing incidence of type 2 DM in young people (6, 7). As a result, there are patients with a long duration of diabetes despite their young age (6, 7). Suboptimal medical attention has been given to patients with young-onset type 2 DM, despite their long disease duration, because of the absence of clinical guidelines targeted at this age group (1-3) and a failure to consider the long diabetes duration, possibly due to a misconception of low risk in these patients. The European Society of Cardiology guidelines take into account the duration of diabetes and categorize patients as high or moderate risk based on diabetes duration (3). However, there is limited existing evidence on the impact of lipid levels on CVD risk according to diabetes duration among populations with type 2 DM.

Most guidelines on type 2 DM management currently recommend that treatment goals should be individualized according to several patient characteristics, such as age, diabetes duration, life expectancy, and the absence/presence of complications and comorbidities (8, 9). In individuals with long-standing diabetes, the benefits of stricter glycemic targets are still unclear, particularly for CVD prevention (10). It remains to be determined whether there is a need to set stricter lipid targets for CVD prevention, especially in people with a long duration of diabetes. The Korea National Health Information Database (NHID) is a large prospective population-based cohort database, with extensive baseline biochemical measures, including fasting blood glucose (FBG) and lipid levels, for approximately 5 million participants (11-13). In the present study, we used data from the Korea NHID to investigate the association of diabetes duration with CVD risk and to examine the relationship between lipid levels and CVD risk over the duration and/or course of diabetes.

## Methods

### Study Population

Among 2 613 026 individuals with diabetes who had undergone health examinations provided by the Korean National Health Insurance Service between January 1, 2015, and December 31, 2016, we excluded individuals aged < 20 years (n = 322), those who had a prior diagnosis of myocardial infarction (MI, n = 67 270) or ischemic stroke (IS, n = 78 114), and those who had any missing variables (n = 70 903). To avoid confounding by preexisting disease and to minimize the possible effects of reverse causality, those individuals with a new diagnosis of MI or IS during the first year of follow-up, or those who died during the first year of follow-up, were also excluded (n = 37 174). Finally, 2 359 243 individuals with type 2 DM (1 415 209 men and 944 034 women) who had their lipid levels monitored at baseline and information about their diabetes duration, were enrolled for analyses and followed up to the date of death or to the end of follow-up (December 31, 2020). The mean follow-up duration was 3.9 ± 0.8 years. The protocol of our study was evaluated and approved by the Institutional Review Board of the Catholic University of Korea, Yeouido St. Mary's Hospital (IRB number: SC23ZISE0054). Anonymous and deidentified information was utilized for analysis and therefore informed consent was not required.

### Diabetes Duration and Progression

The Korea NHID includes all claim data and health screening information, with detailed lifestyle questionnaires and laboratory results. The Korean National Health Screening Program measures FBG every 1 to 2 years, enabling an accurate inception of diabetes. The database contains almost all medical use and medications information, enabling determination of the time point to initiation of antidiabetic drug. The current study included participants who underwent the national health checkup in 2015 to 2016. Type 2 DM was defined according to the International Classification of Disease, 10th Revision (ICD-10) codes E11-14 for type 2 DM, as either the principal diagnosis or the first to fourth additional diagnoses, and the prescription of one antidiabetic drug in each year or FBG level ≥ 126 mg/dL. Subjects were categorized according to the duration of diabetes: new-onset (no previous recorded disease code or history of antidiabetic drug prescription, but with a FBG level ≥ 126 mg/dL at health examination), < 5 years, 5 to 9 years, or ≥10 years.

Diabetes progression can be assessed by the number of oral glucose-lowering drugs (GLDs) or use of insulin (14, 15). Subjects were categorized into 4 groups according to their diabetes treatment modality: (i) no oral GLDs; (ii) 1-2 oral GLDs; (iii) ≥ 3 oral GLDs; or (iv) insulin use ± oral GLDs.

### Covariates

Covariates were based on the data from health examination of the index years (2015-2016) and included age, sex, socioeconomic status, body mass index (BMI; kg/m^2^), current smoking status, alcohol consumption, and systolic/diastolic blood pressure (mmHg). Blood samples for the measurement of serum glucose, creatinine, and lipid levels (total cholesterol, triglyceride, high-density lipoprotein cholesterol [HDL-C], and low-density lipoprotein cholesterol [LDL-C] levels) were drawn after an overnight fast. Non-HDL-C levels were calculated by subtracting the HDL-C level from the total cholesterol level. Dyslipidemia was defined as at least one claim per year under ICD-10 code E78 and the prescription of lipid-lowering medication or a total cholesterol level ≥ 240 mg/dL. Estimated glomerular filtration rate (eGFR) was calculated using the equation from the Modification of Diet in Renal Disease study: eGFR = 175 × serum creatinine^−1.154^ × age^−0.203^ × 0.742 (for women) and chronic kidney disease (CKD) was defined as eGFR < 60 mL/min/1.73 m^2^.

### Study End Points

Newly diagnosed cardiovascular events of MI and IS after a 1-year lag period were defined as the study end points. MI was defined by ICD-10 codes I21 or I22, with more than one diagnosis during hospitalization. IS was defined as the recording of ICD-10 codes I63 or I64 during hospitalization and concomitant brain imaging studies, involving brain magnetic resonance imaging or brain computerized tomography. Participants without MI or IS during their follow-up were considered to have completed the study at the date of their death or at the end of the follow-up period, whichever came first. The study population was followed from baseline to the date of death or cardiovascular events, or until December 31, 2020, whichever came first.

### Statistical Analysis

Statistical analyses were performed using SAS software (version 9.4; SAS Institute, Cary, NC, USA), and a *P* value <.05 was considered significant. The baseline characteristics of the subjects are presented as the mean ± SD or n (%). Subjects were classified into 4 groups according to their diabetes duration (new-onset, < 5 years, 5 to 9 years, or ≥ 10 years). The participants in each diabetes duration group were further divided into 5 groups based on LDL-C levels: < 70, 70-99, 100-129, 130-159, and ≥ 160 mg/dL. In this classification, they were divided into 20 groups based on diabetes duration and LDL-C levels.

We further analyzed the association between lipid levels and CVD risk throughout the diabetes duration by categorizing non-HDL-C levels instead of LDL-C levels. Participants were also divided into the following categories according to non-HDL-C level: < 100 mg/dL, 100-129 mg/dL, 130-159 mg/dL, 160-189 mg/dL, and ≥190 mg/dL. The incidence rate of MI or IS was calculated by dividing the number of incident cases by 1000 person-years. Cox proportional hazards analyses were performed to evaluate the association of LDL-C levels and diabetes duration with incident MI or IS, and hazard ratios (HRs) and 95% CIs were calculated. Model 1 was adjusted for age and sex. Model 2 was further adjusted for smoking status, alcohol intake, BMI, FBG level, hypertension, CKD, dyslipidemia medications, insulin use, and number of oral GLDs ≥ 3. The potential effect modification by age, sex, obesity, and dyslipidemia medications was evaluated using stratified analysis and interaction testing using a likelihood ratio test. Cox regression analysis using restricted cubic splines was also performed to examine the associations of diabetes duration and LDL-C levels with outcomes on a continuous scale.

## Results

### Baseline Characteristics of the Study Subjects

In total, 2 359 243 individuals with type 2 DM were enrolled with 29.8% having new-onset DM, 27.2% with diabetes duration of < 5 years, 19.3% with diabetes duration of 5 to 9 years, and 23.6% with diabetes duration of ≥ 10 years. The general characteristics of study participants according to their diabetes duration are summarized in Table 1. Subjects with a longer duration of diabetes were older, had higher prevalence of hypertension, CKD, and dyslipidemia, and were more likely to be taking medications for dyslipidemia. Subjects with new-onset DM were more likely to be men, current smokers and alcohol drinkers, and had higher FBG levels. The use of antidiabetic medications, including insulin, but excluding metformin, was more prevalent in subjects with a longer duration of diabetes (Table 1).

**Table 1.** Baseline characteristics according to diabetes duration

	New-onset	< 5 years	5-9 years	≥ 10 years
N	704 686 (29.8%)	642 125 (27.2%)	456 330 (19.3%)	556 102 (23.6%)
Age, years	53.5 ± 12.3	58.5 ± 11.3	61.8 ± 10.5	65.2 ± 9.8
Sex, male	488 647 (69.3)	364 986 (56.8)	256 577 (56.2)	304 999 (54.9)
Body mass index, kg/m^2^	25.6 ± 3.7	25.8 ± 3.6	25.3 ± 3.4	24.6 ± 3.2
Smoking				
Nonsmoker	329 311 (46.7)	359 469 (56.0)	265 442 (58.2)	345 144 (62.1)
Ex-smoker	158 930 (22.6)	140 361 (21.9)	101 365 (22.2)	123 102 (22.1)
Current smoker	216 445 (30.7)	142 295 (22.2)	89 523 (19.6)	87 856 (15.8)
Alcohol drinking	94 771 (13.5)	56 347 (8.8)	35 270 (7.7)	33 311 (6.0)
Regular exercise	143 604 (20.4)	134 306 (20.9)	102 129 (22.4)	133 009 (23.9)
Income, lower 25%	145 611 (20.7)	140 132 (21.8)	103 170 (22.6)	117 040 (21.1)
Systolic BP, mmHg	129.4 ± 15.3	127.7 ± 14.6	127.9 ± 14.7	128.5 ± 15.3
Diastolic BP, mmHg	80.33 ± 10.31	78.38 ± 9.66	77.32 ± 9.45	75.75 ± 9.53
Fasting glucose, mg/dL	151.6 ± 39.1	138.2 ± 46.5	140.6 ± 45.1	147.6 ± 51.0
eGFR, mL/min/1.73 m^2^	93.7 ± 60.0	91.8 ± 51.3	88.6 ± 49.4	83.0 ± 47.7
Baseline TC, mg/dL	207.6 ± 41.8	185.0 ± 43.1	174.9 ± 39.1	170.03 ± 38.53
Hypertension	308 882 (43.8)	374 968 (58.4)	294 427 (64.5)	377 754 (67.9)
Chronic kidney disease	31 913 (4.53)	42 015 (6.54)	46 280 (10.14)	99 167 (17.83)
Dyslipidemia	245 584 (34.9)	417 632 (65.0)	293 837 (64.4)	354 110 (63.7)
Dyslipidemia medications	127 996 (18.2)	397 391 (61.9)	283 081 (62.0)	342 812 (61.7)
Pharmacologic therapy for DM				
Insulin	0	42 842 (6.7)	36 714 (8.1)	110 756 (19.9)
Metformin	0	586 401 (91.3)	409 270 (89.7)	486 762 (87.5)
Sulfonylurea	0	178 956 (27.9)	235 033 (51.5)	375 030 (67.4)
DPPIV-inhibitors	0	316 433 (49.3)	265 178 (58.1)	355 087 (63.9)
Thiazolidinedione	0	42 488 (6.6)	51 987 (11.4)	82 422 (14.8)
Alpha-glucosidase inhibitors	0	8374 (1.3)	16 377 (3.6)	38 430 (6.9)
SGLT2 inhibitors	0	25 154 (3.9)	15 638 (3.4)	20 530 (3.7)
Meglitinides	0	1814 (0.3)	2526 (0.6)	6128 (1.1)
GLP-1 RA	0	255 (0.04)	345 (0.08)	980 (0.18)

Values are expressed as mean ± SD, or number (%).

Abbreviations: BP, blood pressure; eGFR, estimated glomerular filtration rate; TC, total cholesterol; DM, Diabetes Mellitus; DPPIV-inhibitors, dipeptidyl-peptidase IV inhibitors; SGLT2 inhibitors, sodium glucose co-transporter 2 inhibitors; GLP-1 RA, glucagon-like peptide-1 receptor agonist.

*P* values for the trend were <.0001 for all variables.

### Diabetes Duration, LDL-C Levels, and the Risk of MI or IS

**Figure 1.**
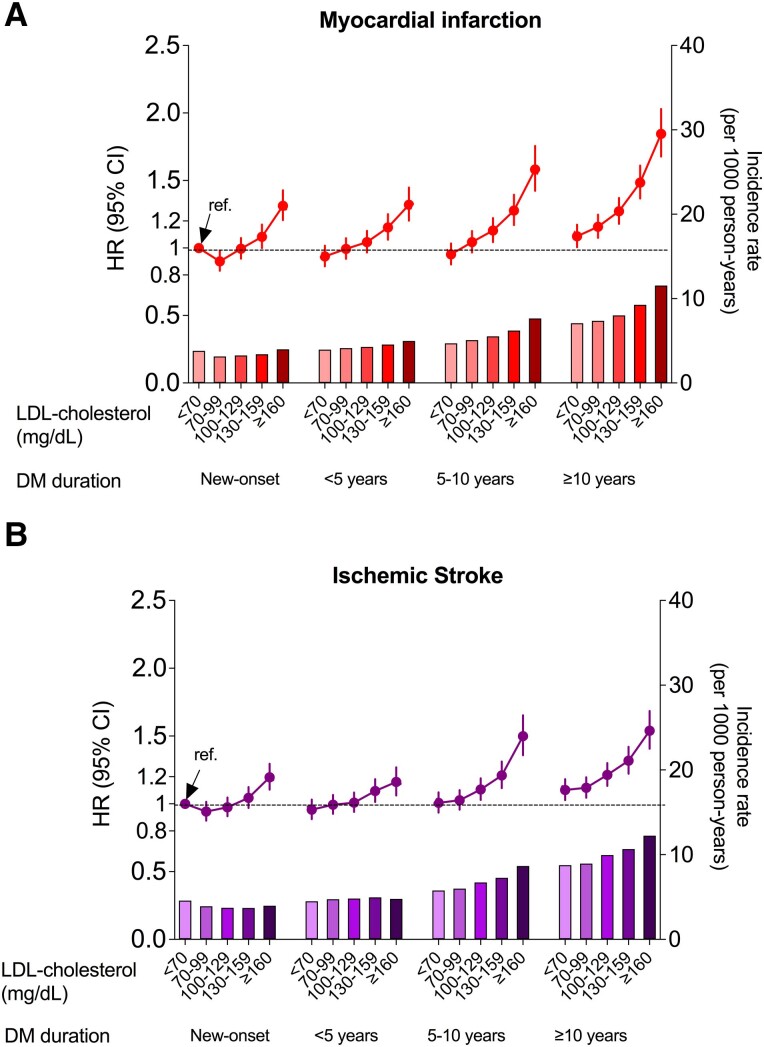
Incidence rate, hazard ratios (HRs), and 95% CIs of myocardial infarction (A) and ischemic stroke (B) according to diabetes duration and low-density lipoprotein cholesterol (LDL-C) levels. The new-onset diabetes group with LDL-C < 70 mg/dL was used as the reference group. Adjusted for age, sex, body mass index, income status, smoking, alcohol drinking, fasting glucose, hypertension, chronic kidney disease, insulin use, dyslipidemia medication, and number of oral glucose-lowering drugs ≥ 3.

During follow-up, 45 883 cases of MI and 53 538 cases of IS were identified. A gradual increase in the incidence rates of MI and IS was observed as the diabetes duration increased (Fig. 1). The associations between diabetes duration and LDL-C levels on a continuous scale and the risk of MI and IS also showed similar patterns with a linear trend (*P* for linearity <.001; Supplementary Fig. S1) (16). We analyzed the new-onset diabetes group with LDL-C < 70 mg/dL as the reference group. The risk of MI or IS began to increase at LDL-C ≥ 160 mg/dL in the new-onset diabetes group, and at LDL-C ≥ 130 mg/dL in the diabetes duration < 5 years group (Tables 2 and 3). Among subjects with diabetes duration 5 to 9 years, LDL-C 100-129 mg/dL, LDL-C 130-159 mg/dL, and ≥ 160 mg/dL were significantly associated with the risk of MI, with HRs (95% CI) of 1.13 (1.04-1.22), 1.28 (1.17-1.39), and 1.58 (1.42-1.76), respectively (Table 2, Fig. 1). The risk of MI in the group with diabetes duration ≥ 10 years was increased by 16%, even in the LDL-C 70-99 mg/dL population (HR [95% CI] = 1.16 [1.08-1.25] for LDL-C 70-99 mg/dL). Among subjects with diabetes duration ≥ 10 years, LDL-C 100-129 mg/dL, LDL-C 130-159 mg/dL, and ≥160 mg/dL were significantly associated with the risk of MI, with HRs (95% CI) of 1.27 (1.18-1.37), 1.48 (1.37-1.61), and 1.85 (1.68-2.03), respectively (Table 2). Similar results were observed for the IS outcome, which displayed relatively attenuated HRs compared with MI (Table 3). Using the new-onset diabetes group with LDL-C < 55 mg/dL as the reference group, the LDL-C cutoffs for increased CVD risk varied depending on the duration of diabetes (data not shown). Similar results were also observed after further adjusting for the presence of peripheral artery disease (data not shown).

**Table 2.** Impact of low-density lipoprotein cholesterol level on myocardial infarction according to diabetes duration

Diabetes duration	LDL-C, mg/dL	N	Events (n)	Incidence rate (per 1000 person-years)	Model 1*^a^*	Model 2*^a^*
New-onset	< 70	58 900	848	3.81	1 (Ref.)	1 (Ref.)
	70-99	154 480	1857	3.15	0.89 (0.82, 0.96)	0.90 (0.83, 0.98)
	100-129	223 557	2788	3.26	0.97 (0.90, 1.05)	0.99 (0.92, 1.07)
	130-159	167 969	2183	3.40	1.06 (0.98, 1.14)	1.08 (1.00, 1.17)
	≥160	99 780	1517	3.99	1.29 (1.18, 1.40)	**1.31 (1.21, 1.43)**
< 5 years	<70	125 485	1918	3.95	0.97 (0.89, 1.05)	0.94 (0.86, 1.02)
	70-99	198 982	3222	4.14	1.02 (0.95, 1.10)	0.99 (0.92, 1.07)
	100-129	170 827	2863	4.27	1.08 (1.00, 1.17)	1.04 (0.97, 1.13)
	130-159	96 050	1723	4.56	1.21 (1.11, 1.31)	**1.15 (1.06, 1.25)**
	≥160	50 781	990	4.98	1.39 (1.27, 1.53)	**1.32 (1.20, 1.45)**
5-9 years	<70	110 808	2008	4.69	1.04 (0.96, 1.12)	0.95 (0.88, 1.03)
	70-99	158 631	3144	5.09	1.12 (1.04, 1.21)	1.04 (0.97, 1.13)
	100-129	115 512	2500	5.53	1.23 (1.14, 1.33)	**1.13 (1.04, 1.22)**
	130-159	51 291	1248	6.21	1.41 (1.29, 1.53)	**1.28 (1.17, 1.39)**
	≥160	20 088	599	7.65	1.80 (1.62, 2.00)	**1.58 (1.42, 1.76)**
≥10 years	<70	150 367	4018	7.08	1.37 (1.27, 1.47)	**1.09 (1.01, 1.17)**
	70-99	196 769	5519	7.36	1.43 (1.33, 1.54)	**1.16 (1.08, 1.25)**
	100-129	133 317	4087	8.01	1.57 (1.46, 1.69)	**1.27 (1.18, 1.37)**
	130-159	55 304	1962	9.26	1.86 (1.72, 2.02)	**1.48 (1.37, 1.61)**
	≥ 160	20 345	889	11.55	2.42 (2.20, 2.66)	**1.85 (1.68, 2.03)**

Model 1 adjusted for age and sex.

Model 2 adjusted for age, sex, smoking status, alcohol intake, body mass index, fasting blood glucose level, hypertension, chronic kidney disease, dyslipidemia medications, insulin use, and number of oral glucose-lowering drugs (GLDs) ≥ 3.

Bold type indicates statistical significance.

Abbreviation: LDL-C, low-density lipoprotein cholesterol.

^*a*^Values are presented as hazard ratio (95% CI) for risk of myocardial infarction.

**Table 3.** Impact of low-density lipoprotein cholesterol (LDL-c) level on ischemic stroke according to diabetes duration

Diabetes duration	LDL-C, mg/dL	N	Events (n)	Incidence rate (per 1000 person-years)	Model 1*^a^*	Model 2*^a^*
New-onset	<70	58 900	1018	4.57	1 (Ref.)	1 (Ref.)
	70-99	154 480	2299	3.90	0.94 (0.87, 1.01)	0.94 (0.88, 1.02)
	100-129	223 557	3189	3.73	0.98 (0.91, 1.05)	0.98 (0.91, 1.05)
	130-159	167 969	2385	3.72	1.04 (0.97, 1.12)	1.04 (0.97, 1.12)
	≥160	99 780	1508	3.97	1.17 (1.08, 1.27)	**1.20 (1.11, 1.30)**
< 5 years	<70	125 485	2187	4.50	0.90 (0.84, 0.97)	0.96 (0.89, 1.03)
	70-99	198 982	3685	4.74	0.95 (0.89, 1.02)	0.99 (0.93, 1.07)
	100-129	170 827	3230	4.82	1.01 (0.94, 1.08)	1.01 (0.94, 1.08)
	130-159	96 050	1873	4.96	1.10 (1.02, 1.19)	**1.10 (1.01, 1.18)**
	≥160	50 781	949	4.77	1.15 (1.06, 1.26)	**1.16 (1.06, 1.27)**
5-9 years	<70	110 808	2463	5.77	1.00 (0.93, 1.08)	1.01 (0.94, 1.09)
	70-99	158 631	3700	6.00	1.04 (0.97, 1.11)	1.03 (0.96, 1.10)
	100-129	115 512	3026	6.71	1.17 (1.09, 1.26)	**1.11 (1.03, 1.19)**
	130-159	51 291	1458	7.27	1.30 (1.20, 1.40)	**1.21 (1.12, 1.31)**
	≥160	20 088	676	8.66	1.63 (1.48, 1.80)	**1.50 (1.36, 1.65)**
≥ 10 years	<70	150 367	4954	8.75	1.26 (1.18, 1.35)	**1.10 (1.03, 1.18)**
	70-99	196 769	6686	8.94	1.29 (1.21, 1.38)	**1.12 (1.05, 1.20)**
	100-129	133 317	5060	9.96	1.45 (1.36, 1.55)	**1.21 (1.13, 1.30)**
	130-159	55 304	2252	10.66	1.61 (1.49, 1.73)	**1.32 (1.22, 1.42)**
	≥160	20 345	940	12.23	1.93 (1.77, 2.11)	**1.54 (1.41, 1.68)**

Model 1 adjusted for age and sex.

Model 2 adjusted for age, sex, smoking status, alcohol intake, body mass index, fasting blood glucose level, hypertension, chronic kidney disease, dyslipidemia medications, insulin use, and number of oral glucose-lowering drugs (GLDs) ≥ 3.

Bold type indicates statistical significance.

Abbreviation: LDL-C, low-density lipoprotein cholesterol.

^a^Values are presented as hazard ratio (95% CI) for risk of ischemic stroke.

Using new-onset diabetes with a non-HDL-C level <100 mg/dL as the reference group, the risk of MI began to increase at non-HDL cholesterol ≥ 160 mg/dL in the group with diabetes duration < 5 years, ≥130 mg/dL in the diabetes duration 5 to 9 years group, and ≥100 mg/dL in the diabetes duration ≥ 10 years group (Supplementary Table S1) (16).

### Diabetes Progression, LDL-C Levels, and the Risk of MI or IS

Diabetes progression status was assessed by the number of oral GLDs or use of insulin. The unmedicated group with LDL-C < 70 mg/dL was analyzed as the reference group. Among unmedicated patients, LDL-C ≥ 160 mg/dL was associated with a 32% increased risk of MI (HR [95% CI] = 1.32 [1.21-1.44]; Supplementary Table S2) (16). Among subjects taking 1 to 2 oral GLDs, LDL-C 100-129 mg/dL, LDL-C 130-159 mg/dL, and ≥160 mg/dL were significantly associated with the risk of MI, with HRs (95% CI) of 1.12 (1.04-1.20), 1.27 (1.18-1.37), and 1.49 (1.36-1.62), respectively (Supplementary Table S2) (16). Among subjects taking ≥ 3 oral GLDs or insulin ± oral GLDs, even LDL-C < 70 mg/dL was associated with an increased risk of MI. Among subjects taking insulin, LDL-C 70-99 mg/dL, LDL-C 100-129 mg/dL, LDL-C 130-159 mg/dL, and ≥160 mg/dL were significantly associated with the risk of MI, with HRs (95% CI) of 1.93 (1.78-2.09), 2.06 (1.90-2.24), 2.27 (2.06-2.49), and 2.72 (2.43-3.05), respectively (Supplementary Table S2) (16). Similar results were observed for the IS outcome, which displayed relatively attenuated HRs compared with MI (Supplementary Table S3) (16).

### Subgroup Analysis

We performed stratified analyses by age, sex, BMI category, and use of dyslipidemia medications, and found that the LDL-C cutoff level for increasing risk of MI or IS varied with the duration of diabetes in all subgroups (Supplementary Tables S4-S7) (16).

Among younger patients (< 65 years), the risk of MI began to increase at LDL-C ≥ 130 mg/dL in the new-onset diabetes and the diabetes duration < 5 years groups, and at LDL-C ≥ 100 mg/dL in the group with diabetes duration 5 to 9 years (Supplementary Table S4) (16). The risk of MI in the group with diabetes duration ≥ 10 years was increased by 13%, even in the LDL-C < 70 mg/dL group and by 20% in the LDL-C 70-99 mg/dL group (HR [95% CI] = 1.13 [1.02-1.26] for LDL <70 mg/dL; and 1.20 [1.08-1.33] for LDL 70-99 mg/dL; Supplementary Table S4) (16). Among patients ≥ 65 years of age, the risk of MI began to increase at LDL-C ≥160 mg/dL in the group with new-onset diabetes and in the group with diabetes duration < 5 years, and it increased at LDL-C ≥ 130 mg/dL in the group with diabetes duration 5 to 9 years. The risk of MI in the group with diabetes duration ≥ 10 years was increased by 19% in the LDL-C 100-129 mg/dL (HR [95% CI] of 1.19 [1.06-1.32] for LDL 100-129 mg/dL; Supplementary Table S4) (16). Higher adjusted HRs for MI or IS were observed in younger patients (< 65 years), men, and the subgroup of patients with obesity (all *P* values for interaction <.05). Higher adjusted HRs for MI, but not IS, were observed in the subgroup without dyslipidemia medication (Supplementary Table S5) (16).

## Discussion

The effect of LDL-C on CVD risk is dependent on the duration of diabetes: the longer the duration, the greater the risk of CVD, even at low levels of LDL-C. Therefore, stricter LDL-C control is needed for those patients with a longer duration of diabetes to prevent CVD. Diabetes duration, as a marker of cumulative exposure to chronic hyperglycemia, should be considered an important risk factor for CVD, relative to the mere presence of DM. LDL-C ≥ 130 mg/dL with diabetes duration < 5 years, LDL-C ≥ 100 mg/dL with diabetes duration 5 to 9 years, and LDL-C ≥ 70 mg/dL with diabetes duration ≥10 years were associated with elevated risk for CVD events, within a large cohort of more than 2 million adults with type 2 DM in Korea.

Previous studies reported that patients diagnosed with early-onset (at 20-39 years of age) type 2 DM had a higher risk of developing CVD and a higher cardiac 10-year expected risk than patients with late-onset type 2 DM (6). These studies suggested that the impending increase in average duration of diabetes in individuals with type 2 DM is likely to increase the burden of CVD (17). A shift in the age at diagnosis to 10 years earlier, equivalent to a 10-year longer duration of diabetes, was associated with a 20% to 30% increased risk of all-cause mortality and a 60% increased risk of CVD mortality (6). In a study conducted in Hong Kong, patients with young-onset type 2 DM, defined by age at diagnosis of < 40 years, had a 30% to 50% higher cumulative risk of CVD than patients with later-onset type 2 DM (7). However, when these results were further adjusted for duration of diabetes, the association of young-onset type 2 DM with CVD events became nonsignificant. Young-onset type 2 DM had higher risks for cardiovascular–renal complications at any given age, driven by longer disease duration. In the Hong Kong cohort, only 28% of the patients with young-onset type 2 DM with dyslipidemia were receiving lipid-lowering agents, and antihypertensive drugs were prescribed to only 48% of those with hypertension (7). Lipid management in type 2 DM requires consideration of the duration of diabetes, regardless of age.

With improvements in interventions to prevent and treat the classic complications of diabetes, people with diabetes are living longer and hence bearing larger CVD complication burdens. In general, duration of diabetes is associated with an increased risk of CVD: a 5-year increase in diabetes duration was associated with an approximately 20% excess risk for MI and stroke (4). Although current recommendations focus on less strict glycemic control with a longer duration of diabetes (8, 9), conversely, there should be more aggressive lipid control under these conditions. Recently, it was reported that the addition of diabetes duration to the classic risk score improved the risk prediction for all CVD outcomes among participants with diabetes (18).

Diabetes progression or severity can also be assessed by the use of insulin or the number of oral GLDs used (14, 15). In our study, we found that there was a markedly increased risk of MI or IS, even with low LDL-C levels, among subjects using insulin or ≥ 3 oral GLDs. While there are arguments that insulin therapy could increase the risk of CVD in type 2 DM, the increased risk of CVD in insulin users and those taking ≥ 3 oral GLDs suggests that insulin use should be interpreted as a marker of diabetes progression or severity. One study found that insulin increased cardiovascular events in a dose-dependent manner: high-dose insulin therapy (≥ 53 U/day) was associated with 3 times higher risk of CVD compared with low exposure (≤ 24.2 U/day). However, these findings need to be interpreted with caution due to the observational nature of the study (19). As seen in patients with type 1 DM in the Diabetes Control and Complications Trial (DCCT), insulin therapy reduced CVD events by improving glycemic control (20). Insulin does not accelerate atherosclerosis and may decrease atherosclerosis by lowering glucose levels, although the protective effects are mainly observed in patients with type 1 DM over a protracted period (20).

Potential mechanisms have been proposed for why a longer duration of diabetes is associated with increased risk of CVD, starting with low LDL-C levels (17, 21-23). Diabetes duration, as a marker of cumulative exposure to chronic hyperglycemia, has been suggested to be linked with atherosclerotic lesions, including intimal–medial thickness and thin cap fibroatheromas (21). Other disorders caused by long-standing hyperglycemia, such as endothelial dysfunction, and abnormalities in fibrinogen and clotting mechanisms may also contribute to the development of vascular complications in patients with DM (22). Diabetes has been shown to be associated with systematic oxidative stress, and prolonged exposure to oxidative stress may represent another mechanism by which CVD risk increases, even with low LDL-C levels (23). Serum oxidized LDL levels increase with the length of diabetes, independent of LDL-C levels, and it is known that oxidized LDL, derived from LDL-C under oxidative stress, possesses many atherogenic properties (23).

Our study findings should be interpreted with several limitations in mind. First, the duration of diabetes was calculated based on the year of type 2 DM diagnosis using the ICD-10 codes and the prescription of one antidiabetic drug; this approach may be somewhat inaccurate, as a lag time exists between onset and diagnosis. In other studies of the duration of diabetes (4, 18), duration was self-reported. This may have resulted in some misclassification, especially for those with longer diabetes duration, as it may have been more challenging to report the age at diagnosis accurately. Second, although we adjusted for several major confounding factors, including smoking, hypertension, and CKD, residual confounding issues may be present. Changes in confounding factors, including diabetes control status, during follow-up were also not included in the analyses because such data were not available. We did not study data on hemoglobin A1c or postprandial glucose levels, since it is difficult to conduct these tests for all participants in a mass screening program. Third, given that our study included only the Korean population, the findings cannot be generalized to other ethnicities. Despite these limitations, the strengths of our study are its large sample size, longitudinal design, and data abundance regarding demographic characteristics, lifestyle factors, and biochemical and comorbidity data. Last, we did not exclude patients with other ischemic heart disease, such as angina, at baseline, and confounding from this may have remained. The diagnosis of other ischemic heart disease can be operationally defined using medical claims records, but there are possibilities of false exclusion or inclusion. However, hospitalization with primary diagnostic ICD-10 codes I21, I22, the criteria used to diagnose MI in this study, had a positive predictive value of 92% (24).

## Conclusion

Rather than simply considering the presence or absence of DM, the risk of CVD should be assessed by considering the duration and the progression of diabetes, and the target goal of lipid levels should change accordingly. In this study, we observed that the LDL-C cutoff level for increasing the risk of CVD varied with the duration of diabetes. The incidence rates and relative risks of CVD were highest in the groups with longer diabetes duration (≥ 10 years) or advanced diabetes, suggesting that screening and preventive treatment for CVD should be intensified with increasing diabetes duration or diabetes progression.

## Data Availability

Original data generated and analyzed during this study are included in this published article or in the data repositories listed in references.

## Abbreviations

BMI: body mass index
CKD: chronic kidney disease
CVD: cardiovascular disease
DM: diabetes mellitus
eGFR: estimated glomerular filtration rate
FBG: fasting blood glucose
GLDs: glucose-lowering drugs
HDL-C: high-density lipoprotein cholesterol
HR: hazard ratio
ICD-10: International Classification of Disease, 10th Revision
IS: ischemic stroke
LDL-C: low-density lipoprotein cholesterol
MI: myocardial infarction
NHID: National Health Information Database (Korea)
